# Psychiatrists’ Knowledge, Attitudes, and Practices Regarding the Use of Modified Electroconvulsive Therapy in Adolescents With Major Depressive Disorder: A Cross-Sectional Survey in Chongqing, China

**DOI:** 10.62641/aep.v54i3.2197

**Published:** 2026-06-15

**Authors:** Jingran Li, Xiwen Tan, Ruishen Li, Ning Du, Yang Zhou, Wo Wang

**Affiliations:** ^1^Department of Psychiatry, University-Town Hospital of Chongqing Medical University, 401331 Chongqing, China; ^2^Department of Clinical Psychology, People’s Hospital of Chongqing Liangjiang New Area, 401121 Chongqing, China

**Keywords:** major depressive disorder, electroconvulsive therapy, adolescent, psychiatrists, health knowledge, practice

## Abstract

**Background:**

Adolescent major depressive disorder (MDD) is a serious condition often resistant to treatment. Despite established efficacy in adults, modified electroconvulsive therapy (MECT) remains limited and controversial in youth. This study examined psychiatrists’ knowledge, attitudes and practices (KAPs) regarding MECT for adolescent MDD, along with their interrelationships and associated factors.

**Methods:**

A cross-sectional online survey was conducted (May–July 2025) amongst psychiatrists in Chongqing, China. Using a validated KAP questionnaire, data were collected via WeChat and examined via descriptive, correlation and regression analyses.

**Results:**

Amongst the 125 questionnaires distributed, 113 valid responses were analysed (90.4%). Psychiatrists showed moderate knowledge (3.31 ± 0.88), positive attitude (3.68 ± 0.51) and moderate practice (3.09 ± 0.98) levels regarding MECT. KAPs were positively correlated. Higher professional level (attending physician: β = 0.374, *p* = 0.007; associate senior or above: β = 0.470, *p* = 0.004) significantly predicted greater knowledge. Better knowledge (β = 0.225, *p* = 0.028) predicted more positive attitude. Higher knowledge (β = 0.496, *p* < 0.001) and attitude (β = 0.226, *p* = 0.006) significantly predicted greater MECT-related practice. Working in a specialized psychiatric hospital (β = –0.525, *p* < 0.001) and clinical experience greater than 20 years (β = 0.324, *p* = 0.030) were significantly associated with practice.

**Conclusions:**

Psychiatrists in Chongqing showed positive attitude but only moderate knowledge and practice regarding MECT for adolescent MDD. Enhancing clinician education and addressing patient- and system-level barriers may help promote appropriate MECT use.

## Introduction

Major depressive disorder (MDD) in adolescence is a prevalent and disabling condition worldwide, affecting approximately 11.0% of adolescents over their lifetime and 7.5% within a 12-month period [1]. Notably, the global burden of MDD has increased substantially amongst individuals aged 10–24 years over the past three decades [2]. Adolescent MDD is consistently associated with a cascade of adverse outcomes such as diminished academic attainment [3], strained peer and family relationships [4], higher risk of substance use [5] and increased suicidal ideation and behaviour [6]. Approximately 30–40% of children and adolescents with MDD fail to respond adequately to first-line therapies (such as psychotherapy and SSRIs), thereby meeting the criteria for treatment-resistant depression, which is a condition linked to worse clinical outcomes and a heightened risk of adverse health consequences [7]. These factors underscore the urgent public health need for effective interventions in adolescent MDD.

As the modern, anesthetised form of electroconvulsive therapy (ECT), modified ECT (MECT) is amongst the most effective treatments for severe depression [8]. In adult MDD, ECT has been associated with response rates of approximately 52%–75%, more rapid symptom relief than pharmacotherapy, reduced suicide risk and decreased rates of rehospitalisation [8, 9]. Clinical guidelines recommend ECT as a first-line treatment in emergency psychiatric conditions, such as psychotic depression, high suicidality, catatonia or acute psychotic exacerbations, and as a second-line option for patients who have not responded adequately to pharmacotherapy [10]. In addition, clinical evidence in adolescents with MDD has shown that adjunctive ECT resulted in greater reductions in suicidal thoughts and depressive symptoms than medication alone, with only transient cognitive side effects [11].

Nevertheless, ECT remains underutilised in young people. International guidelines are cautious: the UK NICE guideline stipulates that ECT ‘should only be considered’ in adolescents with severe, life-threatening or treatment-refractory depression and must be administered only in specialist settings by experienced clinicians [12]. By contrast, U.S. professional bodies permit ECT in adolescents when indicated. For instance, the American Academy of Child and Adolescent Psychiatry states that ECT is approved in the U.S. for patients 13 years and older with severe or treatment-resistant depressive episodes [13]. In practice, however, utilisation rates remain extremely low. A 2023 analysis of the U.S. Kids’ Inpatient Database found that ECT was administered in only 0.03% of paediatric hospitalisations, with MDD being the most common diagnosis [14], and a 16-year nationwide analysis identified only 1870 adolescent hospitalisations involving ECT [15]. A recent systematic review of 41 studies reported ECT response rates of 70–82% for adolescent depression but noted that utilisation remains disproportionately low relative to clinical need [16]. Parental concerns about cognitive side effects further constrain its use [17]. Differences across international guidelines underscore an ongoing debate regarding the safety, efficacy and ethical acceptability of MECT in adolescents.

In China, similar uncertainties prevail. A 2023 nationally representative study of 41 provincial tertiary psychiatric hospitals found that 15.6% of child and adolescent inpatients received ECT, with substantial variation across institutions [18]. An earlier single-centre study reported a higher rate of 42.6% [19], suggesting considerable regional and institutional differences in practice patterns. The 2019 Chinese Expert Consensus on ECT established standardised procedures for ECT broadly, but it lacks specific guidance for adolescent patients [20], possibly leaving clinicians uncertain about appropriate practice. Chinese practice generally restricts ECT to adolescents aged 13 years and older, following careful clinical assessment [20]. However, many Chinese parents express reluctance to consent to ECT for their children, often citing concerns about cognitive side effects (such as memory impairment) and social stigma [18, 21]. This issue is particularly salient given the evidence of an increasing burden of depression and other mental health problems in Chinese adolescents in recent years [22, 23, 24]. Despite these challenges, little is known about how Chinese psychiatrists perceive and utilise ECT in adolescents. To the authors’ knowledge, no previous research has investigated the knowledge, attitudes and practices (KAPs) of psychiatrists regarding adolescent ECT in China.

KAP surveys are a well-established approach in healthcare research for identifying gaps in understanding, beliefs and behaviours related to medical interventions [25]. Assessing psychiatrists’ KAPs can reveal misconceptions and educational needs that may hinder appropriate clinical implementation. Therefore, this study surveyed practicing psychiatrists in Chongqing, China, to examine their KAPs regarding the use of MECT for adolescent MDD and explore their interrelationships and associated factors.

## Methods

### Study Design and Setting

This cross-sectional survey was conducted between May and July 2025 in Chongqing, China. The study targeted psychiatrists practicing in psychiatric or general hospitals.

### Participants and Recruitment

Eligible participants were licensed psychiatrists currently practicing in psychiatric or general hospitals in Chongqing. Questionnaires completed in less than 2 min or containing contradictory, implausible or duplicate responses were excluded from the analysis. The 2 min threshold was established on the basis of pilot testing of the questionnaire (n = 15 psychiatrists), which demonstrated that even the fastest possible attentive completion required approximately 3 min. Therefore, responses completed in less than 2 min were considered indicative of rushed, inattentive or automated (bot) responses and excluded to ensure data quality. Participants were recruited via convenience sampling through WeChat distribution of an online questionnaire. The survey was distributed through multiple professional WeChat groups focused on psychiatric continuing education, clinical case discussions and professional networking in the Chongqing region. These groups included (1) the Chongqing Psychiatric Association affiliated groups, (2) hospital-specific departmental groups from five tertiary hospitals and three secondary hospitals across the city and (3) special-interest groups for early-career psychiatrists and residents. Detailed group distribution information can be found in **Supplementary Table 1**.

### Sample Size and Power Calculation

An a priori sample size calculation was performed using G*Power 3.1 (Heinrich Heine University Düsseldorf, Düsseldorf, North Rhine-Westphalia, Germany) for regression analysis. On the basis of Cohen’s conventions for behavioural sciences, a medium effect size (f^2^ = 0.15) was assumed because this represents a clinically meaningful yet realistic magnitude of association given the exploratory nature of the study and the absence of prior KAP research on this specific topic in China. This effect size is widely used in health sciences research when previous estimates are unavailable, providing adequate power to detect meaningful associations without overly optimistic or conservative assumptions. Moreover, two-tailed α = 0.05, power (1-β) = 0.80 and six predictors were assumed. The assumptions of six predictors were based on a review of literature on factors associated with psychiatrists’ KAPs regarding psychiatric treatments [26]. The minimum required sample size was 98. A total of 125 questionnaires were distributed, and 113 valid responses were obtained (response rate of 90.4%).

### Questionnaire Development, Content and Validation

A self-administered questionnaire comprising 29 items was developed to assess psychiatrists’ demographic characteristics and their KAPs regarding the use of MECT in adolescents with MDD. The questionnaire was originally developed and administered in Chinese. The questionnaire consisted of four sections: (1) demographics (five items), (2) knowledge (eight Likert-type items), (3) attitudes (seven items, five of which were scored on a 5-point Likert scale) and (4) practices (nine items, three of which were scored on a 5-point Likert scale). A priori categories were established on the basis of 5-point Likert scale range (1–5) to facilitate interpretation of the scores, with 3 representing the neutral midpoint. Following established conventions in KAP research [27], the mean scores were categorised as follows: 1.00–2.99 indicated “low” level (reflecting responses below neutral), 3.00–3.99 indicated “moderate” level (reflecting responses between neutral and agree) and 4.00–5.00 indicated “high” level (reflecting agree to strongly agree). For attitude scores, given that higher values represent more favourable views toward MECT, mean scores >3.5 were considered as indicating a “positive” orientation because this threshold reflects an average response above the neutral midpoint toward the favourable end of the scale [28]. These categories were applied consistently when describing the findings in the Results section.

The knowledge section assessed psychiatrists’ self-perceived understanding of MECT indications, contraindications, operational procedures, pre-procedure assessment, management of special situations and recent research progress. The attitude section explored respondents’ general opinions toward MECT use in adolescents, perceived efficacy and side effects and views regarding guideline and record-system development. The practice section examined psychiatrists’ prior training and clinical experience with MECT, and institutional implementation and monitoring practices.

Items were developed on the basis of the 2019 Chinese Expert Consensus on MECT [20], relevant international guidelines and previously published instruments adapted for the local context. The draft questionnaire was developed and refined with the involvement of psychiatrists in the author team. It was further reviewed by an expert panel to ensure content relevance and cultural appropriateness. The authors’ team and the panel included clinicians with expertise in MECT and child and adolescent psychiatry.

The development process followed established methodologies for designing KAP questionnaires, which emphasise a multistage approach that included literature review, expert consultation and pilot testing [29, 30, 31]. Consistent with these recommendations, the questionnaire was systematically developed through (1) comprehensive literature review to identify relevant domains and items, (2) content validation by an expert panel comprising clinicians with expertise in MECT and child and adolescent psychiatry and (3) pilot testing with a small sample of psychiatrists (n = 15) to assess comprehensibility and completion time. This rigorous process ensured that the final instrument possessed strong content validity and was appropriately adapted to the local clinical context.

Each knowledge item was rated on a 5-point Likert scale (1–5), and the overall knowledge score was calculated as the mean of the eight items (range of 1–5). The attitude score was computed as the mean of five Likert-type items (items 1, 4, 5, 6, and 7; range of 1–5). The practice score was derived from three Likert items (items 1, 2, and 4; range of 1–5). Multiple-response items were summarised using descriptive statistics. Only Likert-type items were aggregated to compute the attitude and practice subscale scores. The remaining categorical and multiple-response items (attitude items 2 and 3; practice items 3 and 5–9) were designed to capture clinical contexts and barriers and thus reported descriptively rather than included in score calculations. These items, which included reasons for opposing MECT, estimated proportion of patients receiving MECT, typical number of sessions and perceived barriers, are categorical or multiple response in nature, and they were intended to provide contextual information. Consequently, they are not suitable for calculating mean scores on a Likert scale. The complete questionnaire, along with a detailed explanation of item inclusion and exclusion criteria, is provided in the **Supplementary Material**.

Internal consistency was assessed using Cronbach’s alpha (α = 0.918 for the scored items). Sampling adequacy for factor-analytic procedures was evaluated using the Kaiser–Meyer–Olkin (KMO) measure and Bartlett’s test of sphericity (KMO = 0.898; χ^2^ = 1054.763, df = 78, *p* < 0.001).

### Data Collection and Data Management

Data were collected using Questionnaire Star (an online survey platform) and distributed via WeChat (Tencent Holdings Limited, Shenzhen, Guangdong, China). The detailed questionnaire is provided in the **Supplementary Material**. The online form required responses to all items to minimise missing data and recorded completion time. Data were exported to SPSS (Version 24.0, IBM Corp., Armonk, NY, USA) for analysis and stored on a password-protected institutional drive accessible only to study team members.

### Statistical Analysis

Data were analysed using SPSS (version 24.0). The normality of continuous variables (KAP scores) was assessed using Shapiro–Wilk test. Variables that were approximately normally distributed (Shapiro–Wilk test *p* > 0.05) are presented as mean ± standard deviation (SD). For any variables that violated the normality assumption, median and interquartile range (IQR) would have been reported. Categorical variables were presented as frequencies and percentages. For group comparisons, independent-sample t-tests were used for normally distributed continuous variables, and Mann–Whitney U test was applied for non-normally distributed variables. For bivariate associations, Pearson’s correlation coefficients were calculated for normally distributed variables, and Spearman’s rank correlation coefficients would have been used for non-normally distributed variables. Three separate multiple linear regression analyses were conducted to identify independent predictors of KAP scores. The candidate predictors for each model were selected on the basis of univariate analyses: variables with *p* < 0.20 in bivariate comparisons (t-tests or Pearson’s correlations) were entered into the initial multivariable model. In addition, variables with established conceptual relevance from prior literature were considered, although in this study all such variables already met the *p* < 0.20 criterion and were thus included without forced entry. All statistical tests were two-sided with a significance level of *p* < 0.05. Multicollinearity amongst predictor variables in the regression models was assessed using variance inflation factor (VIF), with VIF values below 5 considered indicative of no significant collinearity concerns. The assumptions of multiple linear regression (linearity, independence of errors, homoscedasticity, normality of residuals, and absence of multicollinearity) were checked and satisfied.

## Results

### Participant Characteristics

A total of 113 psychiatrists were included in the final analysis. Most participants were young and early in their careers, predominantly resident physicians, and slightly more than half were female. The majority worked in psychiatric wards of general hospitals. Detailed demographic characteristics are presented in Table 1.

**Table 1.** **Demographic characteristics of the participants**.

Variable	Category	n (%)
Age	<30 years	68 (60.2)
	31–50 years	38 (33.6)
	>50 years	7 (6.2)
Gender	Male	50 (44.2)
	Female	63 (55.8)
Professional level	Resident physician	76 (67.3)
	Attending physician	27 (23.9)
	Associate senior or above	10 (8.8)
Clinical experience	<5 years	65 (57.5)
	5–10 years	23 (20.4)
	11–20 years	15 (13.3)
	>20 years	10 (8.8)
Workplace	General hospital	71 (62.8)
	Specialized psychiatric hospital	42 (37.2)

### Descriptive Findings of KAP

Normality of the main outcome variables was assessed using Shapiro–Wilk test. The results indicated that knowledge scores (W = 0.979, *p* = 0.082), attitude scores (W = 0.984, *p* = 0.168), and practice scores (W = 0.976, *p* = 0.054) were approximately normally distributed. Therefore, these variables are reported as mean ± SD, and parametric tests (t-tests and Pearson’s correlations) were used for the subsequent analyses. The mean scores and distributions for each domain are presented in Tables 2 and 3. The participants demonstrated moderate knowledge (3.31 ± 0.88) and practice (3.09 ± 0.98) levels, with a positive attitude toward MECT (3.68 ± 0.51). The frequency distributions showed that the knowledge scores were concentrated in the 3.0–3.9 range (58.4%), the attitude scores were predominantly in the 3.5–4.4 range (69.0%) and the practice scores showed a wider distribution with 46.0% in the 2.5–3.4 range.

**Table 2.** **Attitudes-related descriptive findings regarding MECT use in adolescent MDD**.

Item	Response Options	n (%)
A2^*^	High risk of suicide/self-harm/impulsive aggression	106 (93.8)
	Depressive stupor	105 (92.9)
	Poor pharmacotherapy response	94 (83.2)
	Psychotic symptoms	82 (72.6)
A3^*^	Adverse effects (e.g., memory loss)	2 (1.8)
	Long treatment course/poor adherence	2 (1.8)
	Financial burden/limited coverage	1 (0.9)
	Anesthesia risks/safety	1 (0.9)

Note: Table 2 reports non-scored categorical and multiple-response items from the attitudes section. A2: In which situations do you support the use of MECT in adolescents? A3: If you oppose MECT use in adolescents, what are your main reasons? ^*^Indicates multiple-response items; percentages may sum to more than 100%. For the attitude-based item A3, only those respondents who expressed opposition to the implementation of electroconvulsive therapy provided responses for this item, and the sum of percentages may be lower than 100%.

**Table 3.** **Practices-related descriptive findings regarding MECT use in adolescent MDD**.

Item	Response Options	n (%)
P3	<5%	32 (28.3)
	5–15%	22 (19.5)
	15–30%	27 (23.9)
	31–50%	21 (18.6)
	>50%	11 (9.7)
P5	<6 sessions	12 (10.6)
	6–8 sessions	50 (44.3)
	9–10 sessions	39 (34.5)
	11–12 sessions	12 (10.6)
P6	Daily ×3, then every other day	48 (42.5)
	Every other day	34 (30.1)
	Once every 2–3 days	21 (18.6)
	Irregular	7 (6.2)
	Daily	3 (2.6)
P7	No	56 (49.5)
	Uncertain	29 (25.7)
	Yes	28 (24.8)
P8^*^	Unrealistic expectations of rapid effect	92 (81.4)
	Patient/family dissatisfaction with medications	75 (66.4)
	Inadequate understanding of indications	65 (57.5)
	Institutional financial incentives	54 (47.8)
P9^*^	Patient/family refusal	100 (88.5)
	Financial constraints	72 (63.7)
	Limited medical resources	59 (52.2)
	Clinical improvement with other treatments	44 (38.9)

Note: Table 3 reports non-scored categorical and multiple-response items from the practices section. P3: In your estimation, what proportion of eligible adolescents with MDD at your hospital actually receive MECT? P5: What do you consider to be the appropriate number of MECT sessions for first-episode acute-phase adolescent patients with MDD? P6: What is the typical frequency of MECT sessions? P7: Do you believe MECT is overused or misused in clinical practice? P8: If overused, what are the main reasons? P9: What are the main barriers to MECT use in adolescents? ^*^Indicates multiple-response items; percentages may sum to more than 100%.

Item-level analyses further illustrated the psychiatrists’ perspectives and practices regarding MECT. Most respondents supported its use for patients with high suicide risk, depressive stupor or poor pharmacotherapy response. Only a few expressed opposition, mainly due to concerns about adverse effects, anesthesia risks or financial burden. In practice, the psychiatrists reported that MECT was used in a minority of adolescent patients, typically delivered in 6–8 sessions, initially daily then every other day. About one-quarter perceived overuse or misuse, often linked to unrealistic expectations of rapid improvement or dissatisfaction with medications. The most common barriers to indicated use were patient or family refusal and financial limitations (Tables 2 and 3).

### Associations Amongst KAPs and Demographic Factors

Pearson’s correlation analysis showed significant positive associations amongst the three domains: knowledge–attitude (r = 0.286, *p* = 0.002), knowledge–practice (r = 0.594, *p* < 0.001) and attitude–practice (r = 0.299, *p* = 0.001). Higher knowledge of MECT was associated with more favourable attitudes and greater engagement in MECT-related practice. As shown in Table 4, higher knowledge scores were associated with older age, higher professional level and longer clinical experience. The practice scores varied significantly by age, professional level and workplace, whereas the attitude scores showed no significant demographic differences.

**Table 4.** **Comparison of mean knowledge, attitude, and practice scores across demographic subgroups**.

Variable	Category	Knowledge (mean ± SD)	*p*-value	Attitude (mean ± SD)	*p*-value	Practice (mean ± SD)	*p*-value
Age	<30 years	3.08 ± 0.78	0.004	3.66 ± 0.49	0.674	2.98 ± 0.90	0.037
	31–50 years	3.54 ± 0.83		3.72 ± 0.49		3.31 ± 1.02	
	>50 years	3.65 ± 0.95		3.83 ± 0.71		3.86 ± 1.41	
Gender	Male	3.48 ± 0.84	0.055	3.77 ± 0.51	0.095	3.13 ± 1.00	0.757
	Female	3.16 ± 0.89		3.61 ± 0.50		3.07 ± 0.97	
Professional level	Resident physician	3.07 ± 0.77	<0.001	3.66 ± 0.48	0.616	2.94 ± 0.93	<0.001
	Attending physician	3.74 ± 0.92		3.69 ± 0.49		3.40 ± 0.86	
	Associate senior or above	3.89 ± 0.89		3.84 ± 0.67		4.07 ± 1.26	
Clinical experience	<5 years	3.07 ± 0.88	<0.001	3.62 ± 0.46	0.080	2.95 ± 1.05	0.033
	5–10 years	3.38 ± 0.82		3.69 ± 0.54		3.02 ± 0.99	
	11–20 years	4.02 ± 0.76		3.77 ± 0.38		3.38 ± 1.00	
	>20 years	4.15 ± 0.88		3.83 ± 0.43		3.59 ± 0.82	
Workplace	General hospital	3.33 ± 0.92	0.660	3.65 ± 0.53	0.513	3.31 ± 0.93	0.002
	Specialized psychiatric hospital	3.26 ± 0.81		3.72 ± 0.49		2.72 ± 0.96	

### Multiple Linear Regression Analyses

Based on the univariate analyses presented in Table 4, the following variables met the criterion of *p* < 0.20 and were entered as candidate predictors in the regression models: for knowledge, age (*p* = 0.004), gender (*p* = 0.055), professional level (*p* < 0.001) and clinical experience (*p* < 0.001); for attitude, gender (*p* = 0.095) and clinical experience (*p* = 0.080); and for practice, age (*p* = 0.037), professional level (*p* < 0.001), clinical experience (*p* = 0.033) and workplace (*p* = 0.002). In addition, Pearson’s correlation analyses revealed that knowledge was significantly correlated with attitude (r = 0.286, *p* = 0.002) and practice (r = 0.594, *p* < 0.001), and attitude was significantly correlated with practice (r = 0.299, *p* = 0.001). Therefore, knowledge was included as a candidate predictor for attitude and practice models, and attitude was included as a candidate predictor for the practice model, with all correlations meeting the *p* < 0.20 criterion. Three separate multiple linear regression analyses were performed with KAP scores as dependent variables (Tables 5–8). In the first model (Table 6), higher professional level (attending physician and associate senior or above) was significantly associated with greater knowledge, whereas age, gender and clinical experience were not significant predictors. In the second model (Table 7), knowledge was a significant positive predictor of attitude, whereas gender and clinical experience were not significant. In the third model (Table 8), higher knowledge and more favourable attitudes were significant positive predictors of practice; working in a specialized psychiatric hospital was associated with lower practice scores; and clinical experience greater than 20 years was associated with higher practice scores.

**Table 5.** **Variable coding and assignment**.

Variable	Category	Code
Age	<30 years	0 (Reference)
	31–50 years	1
	>50 years	2
Gender	Female	0 (Reference)
	Male	1
Professional level	Resident physician	0 (Reference)
	Attending physician	1
	Associate senior or above	2
Clinical experience	<5 years	0 (Reference)
	5–10 years	1
	11–20 years	2
	>20 years	3
Workplace	General hospital	0 (Reference)
	Specialized psychiatric hospital	1
Knowledge		Continuous variable (score)
Attitude		Continuous variable (score)
Practice		Continuous variable (score)

**Table 6.** **Multiple linear regression models predicting knowledge**.

Independent variable	B	SE	β	t	*p*-value
Age (1)	–0.205	0.252	–0.111	–0.813	0.418
Age (2)	–1.036	0.587	–0.286	–1.764	0.081
Gender (1)	–0.133	0.160	–0.076	–0.833	0.407
Professional level (1)	0.775	0.281	0.374	2.755	0.007
Professional level (2)	1.444	0.487	0.470	2.963	0.004
Clinical experience (1)	0.045	0.264	0.016	0.170	0.866
Clinical experience (2)	0.065	0.234	0.030	0.276	0.783
Clinical experience (3)	0.490	0.268	0.191	1.827	0.071

Note: R^2^ = 0.273, Adjusted R^2^ = 0.217, F = 4.887, *p* < 0.001.

**Table 7.** **Multiple linear regression models predicting attitude**.

Independent variable	B	SE	β	t	*p*-value
Gender (1)	–0.052	0.095	–0.051	–0.543	0.589
Clinical experience (1)	0.018	0.121	0.015	0.150	0.881
Clinical experience (2)	0.025	0.150	0.017	0.164	0.870
Clinical experience (3)	0.288	0.154	0.184	1.864	0.065
Knowledge	0.129	0.058	0.225	2.233	0.028

Note: R^2^ = 0.108, Adjusted R^2^ = 0.066, F = 2.590, *p* = 0.030.

**Table 8.** **Multiple linear regression models predicting practice scores**.

Independent variable	B	SE	β	t	*p*-value
Age (1)	0.262	0.580	0.063	0.451	0.653
Age (2)	0.631	0.605	0.153	1.043	0.299
Professional level (1)	0.191	0.288	0.081	0.665	0.508
Professional level (2)	0.404	0.499	0.115	0.809	0.421
Clinical experience (1)	0.065	0.234	0.030	0.276	0.783
Clinical experience (2)	0.181	0.266	0.062	0.68	0.498
Clinical experience (3)	1.013	0.461	0.324	2.199	0.030
Workplace (1)	–1.638	0.435	–0.525	–3.765	<0.001
Knowledge	0.567	0.097	0.496	5.815	<0.001
Attitude	0.451	0.161	0.226	2.797	0.006

Note: R^2^ = 0.490, Adjusted R^2^ = 0.440, F = 9.815, *p* < 0.001.

Multicollinearity amongst predictor variables was assessed using VIF to ensure the validity of these regression models. All VIF values were well below the conservative threshold of 5 (knowledge model: range of 1.189–3.768; attitude model: range of 1.077–1.252; practice model: range of 1.309–4.282), indicating that multicollinearity did not adversely affect the regression estimates.

## Discussion

This study aimed to assess psychiatrists’ KAPs regarding MECT for adolescent MDD in Chongqing, China. The findings indicated that psychiatrists demonstrated moderate knowledge and practice levels and generally positive attitudes toward MECT. Most respondents endorsed its use for severe clinical situations such as high suicide risk or depressive stupor, whereas only a few expressed opposition, citing concerns about adverse effects, treatment burden, anaesthesia or cost. Knowledge was positively associated with attitudes and practices, suggesting that greater understanding of MECT principles may translate into more favourable views and increased clinical utilisation. Overall, these results suggest that MECT is recognised as an effective option for treatment-resistant or life-threatening adolescent depression, although its actual clinical implementation remains constrained by patient or family reluctance and financial barriers.

In more detail, the respondents demonstrated a moderate grasp of MECT principles. This level is higher than the “nil or negligible” self-rated knowledge reported by many Belgian child psychiatrists [32], but it still leaves room for improvement. This discrepancy likely reflects differences in training systems and clinical guidelines: Belgian psychiatrists reported minimal ECT training during residency, and Belgium’s restrictive national guidelines limit ECT to “exceptional cases” in adolescents, contributing to clinical unfamiliarity. By contrast, ECT remains an integral component of psychiatric training in many Chinese teaching hospitals, and the 2019 Chinese Expert Consensus provides a standardised framework for practice [20]. Attitudes were overall positive: a large majority viewed ECT as effective and lifesaving. For example, 93.8% endorsed MECT for suicidal adolescents and 92.9% for stuporous depression, mirroring the consensus that ECT can be life-saving in refractory adolescent cases [33]. Only two psychiatrists expressed opposition to MECT for adolescent MDD, citing concerns about side effects, treatment burden, anaesthesia risks and cost. Such reservations may reflect persistent stigma or misconceptions about ECT [34]. Safety perceptions were similarly good: most psychiatrists did not consider MECT cruel or outdated. These findings align closely with a 2025 study of Iranian psychiatric trainees, which reported that 96.5% had received ECT training and 77.4% had administered ECT to 10 or more patients during training, with 86.2% affirming ECT’s effectiveness [35]. The parallel findings between Chinese and Iranian psychiatrists may reflect shared characteristics of ECT utilisation in non-Western contexts, where the procedure remains more routinely integrated into psychiatric practice than in some Western countries. However, only 55.5% of Iranian respondents were familiar with national ECT guidelines, potentially explaining the slightly higher knowledge scores observed in the present study, where the 2019 Chinese Expert Consensus provides clear guidance [20]. These findings align with surveys of psychiatric trainees in Iran, where similarly high proportions affirmed ECT’s efficacy and safety [35]. Thus, in Chinese and Iranian psychiatric samples, ECT is viewed favourably amongst specialists.

Regarding clinical practice, reported MECT use in adolescents was relatively low. Nearly half of respondents believed that fewer than 15% of adolescents with MDD in their hospitals ever received MECT. Most psychiatrists described a typical course of around 6–8 sessions, usually given daily at first and then every other day. About one-quarter felt that MECT was sometimes overused or misused in practice, mainly due to unrealistic expectations of rapid improvement and dissatisfaction with medication outcomes. These perceptions echo observations elsewhere that ECT is sometimes applied eagerly as a “last resort” when rapid change is anticipated [32]. The most common barriers to using MECT despite indication were family or patient refusal and cost. Such barriers are well-known: stigma and misinformation about ECT (often fuelled by negative media portrayals) can lead families to refuse it [34], and financial or logistical constraints impede access. Cross-cultural comparisons further illuminate these findings. In Saudi Arabia, Almughais *et al*. [36]. reported that younger healthcare providers (aged 19–25 years) had better ECT knowledge than older ones, opposite to the finding in the present study that older, more experienced clinicians demonstrated superior knowledge. This discrepancy may reflect differences in the timing of ECT’s introduction into medical curricula: in rapidly developing healthcare systems, younger cohorts may have received more systematic ECT education if the procedure was only recently integrated into training programmes. Furthermore, cultural factors may influence attitudes because stigma and misconceptions about ECT vary across Middle Eastern and Asian contexts, with family refusal often cited as a barrier in both regions [37]. In sum, whilst psychiatrists recognise appropriate indications for adolescent MECT and generally support its use, they report that only a minority of eligible patients actually receive it in their institutions, partly due to external obstacles.

Consistent with KAP theory, positive correlations were found amongst the three domains. Greater MECT knowledge was weakly but significantly associated with more favourable attitudes and moderately strongly associated with more frequent MECT practice. Attitudes were positively and significantly correlated with practice. This pattern of better-informed clinicians have more positive beliefs and act more matches the classic KAP model, which holds that knowledge shapes attitudes and behaviours [38]. For example, scientific reports on other health fields similarly showed that higher knowledge leads to better attitudes and care practices [39].

The knowledge and practice scores varied by clinician demographics. Older clinicians (>30 years), attending-level psychiatrists and those with >5 years of experience had higher knowledge than younger, less experienced colleagues. Similarly, the practice scores were significantly higher amongst older and attending-level clinicians and those working in general hospitals than in those working in specialized psychiatric hospitals. As expected, this finding suggests that advanced training and time in the field translate to increased familiarity with MECT. In fact, an Iranian study found that early-career psychiatrists (with more training) had higher confidence in their ECT knowledge than residents [35]. By contrast, the attitudes toward MECT did not differ across any demographic subgroup in the sample because age, gender, experience, professional level and workplace had no significant effect on how psychiatrists viewed MECT. This finding is in line with those of other works. For instance, Almughais *et al*. [36] reported that gender did not influence ECT knowledge or attitudes amongst Saudi healthcare providers. Notably, Almughais *et al*. [36]. also reported that younger providers (aged 19–25 years) had better ECT knowledge than older ones. Meanwhile, the present study showed the opposite, and cultural or training differences may explain this discrepancy. In general, the findings indicated that beyond knowledge differences by seniority, psychiatrists share similarly positive attitudes toward MECT regardless of background, and those with more knowledge tend to put it into practice.

The regression analyses identified factors that independently predict each domain. For knowledge, professional level remained significant: attending physicians and those with associate senior level or above had significantly higher knowledge than resident physicians. This finding echoes the bivariate results and prior literature showing that advanced training yields enhanced expertise. For attitudes, knowledge was the sole positive predictor, reinforcing the idea that informed clinicians adopt more favourable views. Finally, for practice, the strongest positive predictors were knowledge and attitude, again reflecting the linkage proposed in the KAP framework [38, 39]. By contrast, working in a specialized psychiatric hospital was associated with lower practice scores, and clinical experience greater than 20 years was associated with higher practice scores, with the latter being intuitive (less experience → less practice).

The finding that psychiatrists in specialized psychiatric hospitals reported lower MECT practice scores is particularly noteworthy and may reflect several characteristics of China’s mental health system. Firstly, patients in specialized hospitals often present with greater illness severity and higher rates of involuntary admission than those in general hospital psychiatry departments [40]. Whilst such patients may clinically warrant MECT, the presence of severe behavioural disturbances or involuntary status may lead clinicians to adopt more conservative approaches. Secondly, the national policy has increasingly emphasised the integration of mental health services into general healthcare, with general hospitals positioned as accessible entry points for care [41]. Thus, psychiatrists in general hospitals may encounter a broader range of patients and utilise MECT more readily as part of integrated care models. Thirdly, resource allocation differs: general hospitals typically have established anaesthesia departments, facilitating MECT delivery, whereas some specialized psychiatric hospitals historically faced challenges in accessing anaesthesiologists and perioperative support [42]. Fourthly, institutional treatment philosophies may vary, with evidence suggesting that urban and rural healthcare settings demonstrate different MECT utilisation patterns, which may reflect broadened differences in institutional practices and patient populations [43]. These factors likely interact and warrant further investigation to elucidate the mechanisms underlying this association.

A notable detail that the regression model for knowledge explained 27.3% of the variance (R^2^ = 0.273), indicating that a substantial portion of the variability in psychiatrists’ knowledge about MECT remains unexplained by the demographic and professional factors included in the analysis. This finding suggests that other unmeasured variables may play important roles in shaping knowledge levels. Potential factors not captured in this study include prior exposure to formal MECT training during residency or continuing medical education, personal reading habits and access to academic journals, institutional protocols and educational resources and direct clinical experience with MECT procedures. Future research should explore these additional determinants to better understand what drives knowledge acquisition in this area. Moreover, the relatively low explanatory power of the knowledge model underscores the complexity of knowledge formation and the need for multifaceted educational interventions.

The findings of this study carry several practical implications for clinical training and health policy. Given that knowledge positively predicted attitudes and practices, strengthening MECT-related education is warranted. Residency programmes should consider integrating structured curricula that cover indications, contraindications, procedural details and communication strategies for discussing MECT with families. Continuing medical education initiatives could update practicing psychiatrists on the 2019 Chinese Expert Consensus and address identified knowledge gaps such as the transient nature of cognitive side effects. Public awareness campaigns need to disseminate accurate information about MECT’s safety and efficacy in adolescents to reduce family refusal, which was the most commonly cited barrier, thereby countering stigma and misconceptions. At the policy level, policymakers should evaluate including MECT for treatment-resistant adolescent depression in basic medical insurance schemes to alleviate financial burdens on families. Furthermore, the observed practice differences between general and specialized hospitals highlight the need for standardised clinical protocols and referral pathways to ensure equitable access across healthcare settings. Future research should assess the effect of such interventions and explore regional variations in KAPs to inform national guidelines.

Limitations: Several limitations should be considered. Firstly, the cross-sectional design precludes causal inference. Secondly, the use of convenience sampling via WeChat may have introduced selection bias; the over-representation of junior clinicians (60.2% under 30 years old) suggests that the findings may not fully capture the perspectives of more senior psychiatrists. Thirdly, the sample was restricted to psychiatrists in Chongqing, thereby limiting the generalisability of the findings to other regions with different healthcare systems. Fourthly, self-reported data are subject to social desirability and recall biases. Fifthly, although the newly developed questionnaire demonstrated good internal consistency (Cronbach’s α = 0.918), measurement errors cannot be ruled out. Finally, whilst the sample size was adequate for the primary analyses, it may limit the detection of smaller effects or detailed subgroup analyses. Caution is thus warranted when extrapolating these findings to the national level.

## Conclusions

Psychiatrists in Chongqing showed overall positive attitudes toward MECT for adolescent MDD and moderate knowledge and practice levels. Greater knowledge significantly predicted favourable attitudes and actual MECT use, highlighting the importance of clinician education. Given that guidelines support ECT for severe adolescent depression, efforts to enhance psychiatrists’ understanding and comfort with MECT are warranted. Addressing patient- and system-level barriers (e.g., stigma, family concerns and costs) is equally important because these were reported as major obstacles.

## Availability of Data and Materials

The data used and/or analyzed during the current study are available from the corresponding author upon reasonable request.

## Supplementary Material

Supplementary material associated with this article can be found, in the online version, at https://doi.org/10.62641/aep.v54i3.2197.
