# Supplementary material for: Psychiatrists’ Knowledge, Attitudes, and Practices Regarding the Use of Modified Electroconvulsive Therapy in Adolescents With Major Depressive Disorder: A Cross-Sectional Survey in Chongqing, China
Source: Actas Esp Psiquiatr. 2026 Jun 15;54(3):680–91. doi: 10.62641/aep.v54i3.2197 (PMC13294760; doi:10.62641/aep.v54i3.2197)
Supplement: Supplementary file 1 [file ActEsp-54-3-680-691-s1.zip › Supplementary Material (2).docx]

**Table S1. Distribution of respondents by hospital characteristics and sub-specialty interests based on WeChat group categories**

| **Variable** | **Category** | **n (%)** |
| --- | --- | --- |
| Hospital location | Urban | 89 (78.8) |
|  | Suburban/rural | 24 (21.2) |
| Hospital tier | Tertiary hospitals | 95 (84.1%） |
|  | Secondary hospitals | 18 (15.9%) |
| Sub-specialty interest | Child and adolescent psychiatry | 27 (23.9%) |
|  | Adult psychiatry | 69 (61.1%) |
|  | Other psychiatric sub-specialties | 17 (15.0%) |
