# Supplementary material for: Psychiatrists’ Knowledge, Attitudes, and Practices Regarding the Use of Modified Electroconvulsive Therapy in Adolescents With Major Depressive Disorder: A Cross-Sectional Survey in Chongqing, China
Source: Actas Esp Psiquiatr. 2026 Jun 15;54(3):680–91. doi: 10.62641/aep.v54i3.2197 (PMC13294760; doi:10.62641/aep.v54i3.2197)
Supplement: Supplementary file 1 [file ActEsp-54-3-680-691-s1.zip › Supplementary Material.docx]

**Questionnaire on Psychiatrists' Knowledge, Attitudes, and Practices Regarding MECT for Adolescent MDD**

**Section 1: Demographics**

| **No.** | **Question** | **Options** |
| --- | --- | --- |
| D1 | Age | □ <30 years □ 31-50 years □ >50 years |
| D2 | Gender | □ Male □ Female |
| D3 | Professional level | □ Resident physician □ Attending physician □ Associate senior or above |
| D4 | Clinical experience | □ <5 years □ 5-10 years □ 11-20 years □ >20 years |
| D5 | Workplace type | □ General hospital □ Specialized psychiatric hospital |

**Section 2: Knowledge**

Please rate your level of agreement:1 = Strongly disagree, 2 = Disagree, 3 = Neutral, 4 = Agree, 5 = Strongly agree.

| **No.** | **Item** | **1** | **2** | **3** | **4** | **5** |
| --- | --- | --- | --- | --- | --- | --- |
| K1 | I am familiar with the indications for MECT in adolescents with MDD | □ | □ | □ | □ | □ |
| K2 | I am familiar with the contraindications for MECT in adolescents with MDD | □ | □ | □ | □ | □ |
| K3 | I understand the procedural steps of MECT | □ | □ | □ | □ | □ |
| K4 | I am familiar with pre-MECT assessment requirements | □ | □ | □ | □ | □ |
| K5 | I understand how to manage special situations during MECT (e.g., prolonged seizure, delayed emergence) | □ | □ | □ | □ | □ |
| K6 | I understand the short- and long-term cognitive effects of MECT in adolescents | □ | □ | □ | □ | □ |
| K7 | I follow recent research progress on MECT use in adolescents | □ | □ | □ | □ | □ |
| K8 | I am familiar with the 2019 Chinese Expert Consensus on MECT regarding adolescent patients | □ | □ | □ | □ | □ |

Note: All knowledge items (K1-K8) are 5-point Likert scale items used to calculate the total knowledge score (mean), range 1-5.

**Section 3: Attitudes**

A1, A3, A4, A5, A6 are 5-point Likert scale items: 1 = Strongly disagree, 2 = Disagree, 3 = Neutral, 4 = Agree, 5 = Strongly agree. A2 and A7 are multiple-response items.

| **No.** | **Item** | **1** | **2** | **3** | **4** | **5** |
| --- | --- | --- | --- | --- | --- | --- |
| A1 | MECT is effective for treatment-resistant depression in adolescents | □ | □ | □ | □ | □ |
| A2* | ^*^In which situations do you support the use of MECT in adolescents? | □ Poor pharmacotherapy response □ High risk of suicide/self-harm/impulsive aggression □ Depressive stupor □ Psychotic symptoms | | | | |
| A3* | ^*^If you oppose MECT use in adolescents, what are your main reasons? | □ Financial burden/limited coverage □ Long treatment course/poor adherence □ Adverse effects (e.g., memory loss) □ Anesthesia risks/safety concerns | | | | |
| A4 | The safety profile of MECT in adolescents is acceptable | □ | □ | □ | □ | □ |
| A5 | MECT is necessary for adolescents with high suicide risk | □ | □ | □ | □ | □ |
| A6 | Specific clinical guidelines for adolescent MECT should be developed | □ | □ | □ | □ | □ |
| A7 | Establishing a registry system for adolescent MECT would improve clinical practice |  |  |  |  |  |

Note: Attitude score is calculated as the mean of A1, A4, A5, A6, A7 (range 1-5). A2 and A3 are for descriptive analysis only. ^*^Indicates multiple-response items.

**Section 4: Practices**

P1, P2, P4 are 5-point Likert scale items: 1 = Never, 2 = Rarely, 3 = Sometimes, 4 = Often, 5 = Very frequently. P3, P5-P9 are multiple-response or open-ended items.

| **No.** | **Item** | **1** | **2** | **3** | **4** | **5** |
| --- | --- | --- | --- | --- | --- | --- |
| P1 | How often do you recommend MECT for eligible adolescents with MDD in your clinical practice? | □ | □ | □ | □ | □ |
| P2 | How often is MECT performed on adolescents at your hospital? | □ | □ | □ | □ | □ |
| P3 | In your estimation, what proportion of eligible adolescents with MDD at your hospital actually receive MECT? | □ < 5% □ 5–15%  □ 15-30% □ 31-50% □ >50% | | | | |
| P4 | To what extent do you discuss the risks and benefits of MECT with families before recommending it? | □ | □ | □ | □ | □ |
| P5 | What do you consider to be the appropriate number of MECT sessions for first-episode acute-phase adolescent patients with MDD? | □ <6 sessions □ 6-8 sessions □ 9-10 sessions □ >11-12 sessions | | | | |
| P6 | What is the typical frequency of MECT sessions? | □ Daily ×3, then every other day □ Every other day □ Once every 2-3 days □ Irregular □ Daily | | | | |
| P7 | Do you believe MECT is overused or misused in clinical practice? | □ Yes □ No □ Uncertain | | | | |
| P8* | If overused, what are the main reasons? | □ Unrealistic expectations of rapid effect □ Patient/family dissatisfaction with medications □ Inadequate understanding of indications □ Institutional financial incentives | | | | |
| P9* | *What are the main barriers to MECT use in adolescents? | □ Patient/family refusal □ Financial constraints □ Limited medical resources □ Clinical improvement with other treatments | | | | |

Note: Practice score is calculated as the mean of P1, P2, P4 (range 1-5). P3 and P5-P9 are for descriptive analysis only. ^*^Indicates multiple-response items.
